# QSPR Modeling of Fungicides Using Topological Descriptors

**DOI:** 10.1155/2023/9625588

**Published:** 2023-09-30

**Authors:** Saima Parveen, Fatima Saeed, Fozia Bashir Farooq, Nusrat Parveen, Nazeran Idrees, Sumiya Nasir, Rakotondrajao Fanja

**Affiliations:** ^1^Government College University Faisalabad, Faisalabad 38000, Pakistan; ^2^Imam Muhammad Ibn Saud Islamic University, Riyadh, Saudi Arabia; ^3^Prince Mohammad Bin Fahd University, Khobar 31952, Saudi Arabia; ^4^Antananarivo University, Antananarivo, Madagascar

## Abstract

A topological index is a real number that is obtained from a chemical graph's structure. Determining the physiochemical and biological characteristics of a variety of medications is useful since it more accurately represents the theoretical characteristics of organic molecules. This is accomplished using degree-based topological indices. The QSPR research has improved the structural understanding of the physiochemical properties of fungicides. Thirteen fungicides are examined for some of their physiochemical properties, and a QSPR model is built using nine of the drugs' topological indices. Here, we examine the degree to which the topological indices and physiochemical attributes are connected. To do this, we create networks connecting each of the topological indices to the properties of fungicides and computationally construct topological indices of the drugs mentioned above. According to this QSPR model, the melting point, boiling point, flash point, complexity, surface tension, etc. of fungicides are strongly connected. It was discovered that the topological indices (TIs) applied to the fungicides more accurately represent their theoretical features and show a strong correlation with their physical attributes.

## 1. Introduction

For many decades, fungicides have predominantly been used to control fungal-caused plant diseases that threaten human health and crop production [1]. Losses in crops reached almost one billion dollars. The pathogen (fungus) is highly aggressive under field conditions when the environmental conditions favor the disease development [2]. Currently, due to the unavailability of cultivars with complete resistance, the application of fungicides is the main recommended tool for disease control along with cultural practices [3].

There are presently nine and forty seven groups of contact fungicides with multisite and single-site modes of action, respectively. Single-site active fungicides are less toxic to nontarget organisms. Modern systemic fungicides are typified by the triazoles. This group of fungicides is still the basis of cereal disease management strategies worldwide. Their antifungal activity is based on their ability to inhibit CYP51 (lanosterol 14-demethylase), a key enzyme for sterol biosynthesis in fungi [4]. Each triazole substance may have a somewhat different effect on the metabolic process that produces sterols [5], while the outcomes—abnormal fungal growth and death—are identical in different fungi. Triazole chemicals are crucial because of their outstanding antifungal effectiveness, comparatively low risk of resistance, and long-term stability in soil and water [1]. Triazoles can be used as early infection treatments or as a preventative measure. Some triazole fungicides have antisporulant qualities. However, these are ineffective once a fungus starts to develop spores as spores have enough sterol to form germ tubes. Within the triazole family, the principal compounds are difenoconazole, fenbuconazole, tebuconazole, cyproconazole, myclobutanil, penconazole, propiconazole, tetraconazole, triadimenol, prothioconazole, triticonazole, bromuconazole, epoxiconazole, fluquinconazole, flutriafol, ipconazole, metconazole, paclobutrazol, flusilazole, bitertanol, and triadimefon [6].

Topological indices (TIs) are quantitative descriptors obtained from a chemical graph that thoroughly characterize the chemical system and are widely employed in the study on the physiochemical features of numerous drugs. The chemical graph theory makes extensive use of polynomials and TIs, which are extensively used to depict the chemical structure. Graph invariants (TIs) have recently attracted a lot of attention in studies of quantitative structure-property relationships (QSPRs) and quantitative structure-activity relationships (QSARs) and are used in a wide range of mathematical fields, including bioinformatics, mathematics, informatics, and biology. For further study on QSPR modeling on certain drugs, we encourage readers to read [7–10].

We examined some of the physiochemical characteristics of thirteen fungus therapy medications and created a QSPR model utilizing nine topological indices. For this, we compute topological indices of the drugs analytically and depict graphs relating each of these topological indices to the characteristics of fungus drugs. The melting point, boiling point, flash point, complexity, surface tension, etc. of fungus medicines are closely related according to this QSPR model.

## 2. Preliminaries

In drug configuration, atoms depict vertices, and the associated bonds connecting the atoms are termed as edges. Graph *G*(*V*, *E*) is thought to be simple, finite, and connected, whereas *V* and *E* in the chemical graph are referred to as vertex and the edge set, respectively. The degree of a vertex *u* in the graph *G* is the number of vertices adjacent to *u* in *G* is denoted by *d*_*u*_. In chemistry, the valence of a compound and the degree of a vertex in a graph are concepts that are inextricably linked [11, 12]. The inspiration for this article comes from the idea that different medications (structures) may be identified, and that when they are examined for various factors while keeping topological indices in mind, their dominance can be rated. The QSPR model has been applied for the 9 topological indices, which are given in the following.

**Definition 1 .**

The ABC index [13] is given under

(1) $$\begin{array}{c} \text{ABC}\left(G\right)=\underset{uv\in E\left(G\right)}{\sum }\sqrt{\frac{d_{u+}d_{v}-2}{d_{u}d_{v}}}\text{.} \end{array}$$

**Definition 2 .**

The first degree-based TI is Randic index RA(*G*) calculated by Milan Randic in 1975 [14] is given under

(2) $$\begin{array}{c} \text{RA}\left(G\right)=\underset{uv\in E\left(G\right)}{\sum }\sqrt{\frac{1}{d_{u}d_{v}}}\text{.} \end{array}$$

**Definition 3 .**

The sum connectivity index [15] is given under

(3) $$\begin{array}{c} S\left(G\right)=\underset{uv\in E\left(G\right)}{\sum }\sqrt{\frac{1}{d_{u}+d_{v}}}\text{.} \end{array}$$

**Definition 4 .**

The GA index [16] is given under

(4) $$\begin{array}{c} \text{GA}\left(G\right)=\underset{uv\in E\left(G\right)}{\sum }\frac{2\sqrt{d_{u}d_{v}}}{d_{u}+d_{v}}\text{.} \end{array}$$

**Definition 5 .**

First and second Zagreb indices [17] are given under

(5) $$\begin{array}{c} M_{1}\left(G\right)=\underset{uv\in E\left(G\right)}{\sum }\left(d_{u}+d_{v}\right)\text{,}\\ M_{2}\left(G\right)=\underset{uv\in E\left(G\right)}{\sum }\left(d_{u}d_{v}\right)\text{.} \end{array}$$

**Definition 6 .**

Harmonic index [18] of *G* is given under

(6) $$\begin{array}{c} H\left(G\right)=\underset{uv\in E\left(G\right)}{\sum }\frac{2}{d_{u}+d_{v}}. \end{array}$$

**Definition 7 .**

Hyper Zagreb index [12] is defined as

(7) $$\begin{array}{c} \text{HM}\left(G\right)=\underset{uv\in E\left(G\right)}{\sum }{\left(d_{u}+d_{v}\right)}^{2}\text{.} \end{array}$$

**Definition 8 .**

Forgotten index [16] is given under

(8) $$\begin{array}{c} F\left(G\right)=\underset{uv\in E\left(G\right)}{\sum }\left[{\left(d_{u}\right)}^{2}+{\left(d_{v}\right)}^{2}\right]\text{.} \end{array}$$

## 3. Quantitative Structure Analysis and Regression Model

**Figure 1:**
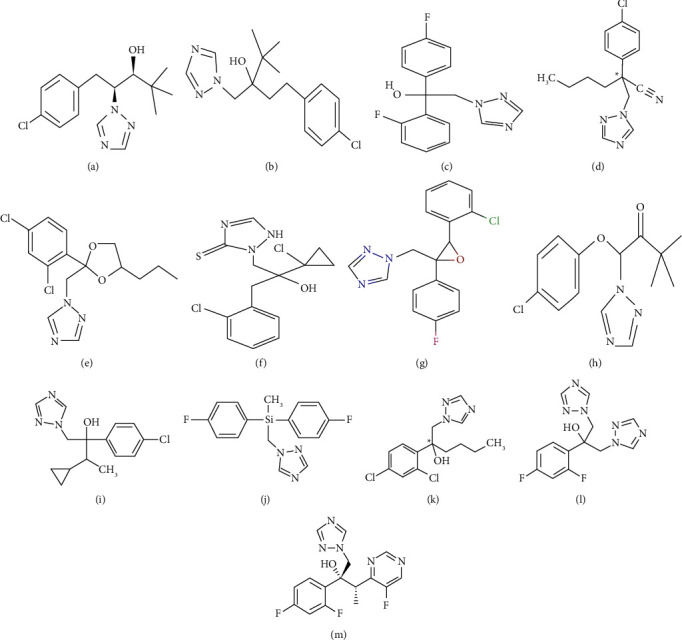
Chemical structure of drugs. (a) Paclobutrazol. (b) Tebuconazole. (c) Flutriafol. (d) Myclobutanil. (e) Propiconazole. (f) Prothioconazole. (g) Epoxiconazole. (h) Triadimefon. (i) cyproconazole. (j) Flusilazole. (k) Hexaconazole. (l) Flucanozole. (m) Voriconazole.

In this section, TIs of the fungicides are computed. The relationship between QSPR analysis and TIs suggests that the physiochemical characteristics of the fungus are highly connected. Thirteen medicines are used in the analysis. The drug edifices are exhibited in Figure 1. We implement regression analysis calculations for this study. Drug computable structure analysis of nine TIs for QSPR modeling tenacity is performed. The topological indices of the respective drugs are computed in Table 1. The ten physical properties, such as solubility in water, boiling point (BP), density, melting point (MP), molar mass, flash point (FP), topological polar surface area, heavy atom count, complexity, and refractive index, are listed in Table 2. We impose a linear model by using the following equation:

(9) $$\begin{array}{c} P=\alpha +\beta \;\left(\text{TI}\right)\text{.} \end{array}$$

**Figure 2:**
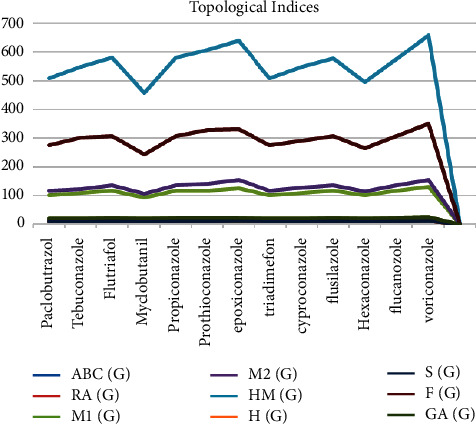
2D graph of medicines with TIs.

**Figure 3:**
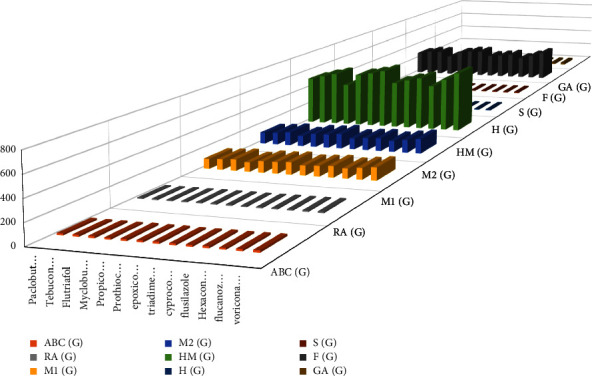
3D graph of medicines with TIs.

*P* denotes the physiochemical property of the given drug. TI stands for topological index, *α* stands for constant, and *β* stands for regression coefficient. MATLAB and R-language software are helpful for results. Linear models are used to analyze nine TIs of the fungicides and their properties. ChemSpider and PubChem are used to get the information given in Table 2. The 2D and 3D graphs of the medicines with TIs are given in Figures 2 and 3, respectively.

### 3.1. Regression Models and Statistical Parameters Comparison between TIs and Correlation Coefficient of Properties

**Figure 4:**
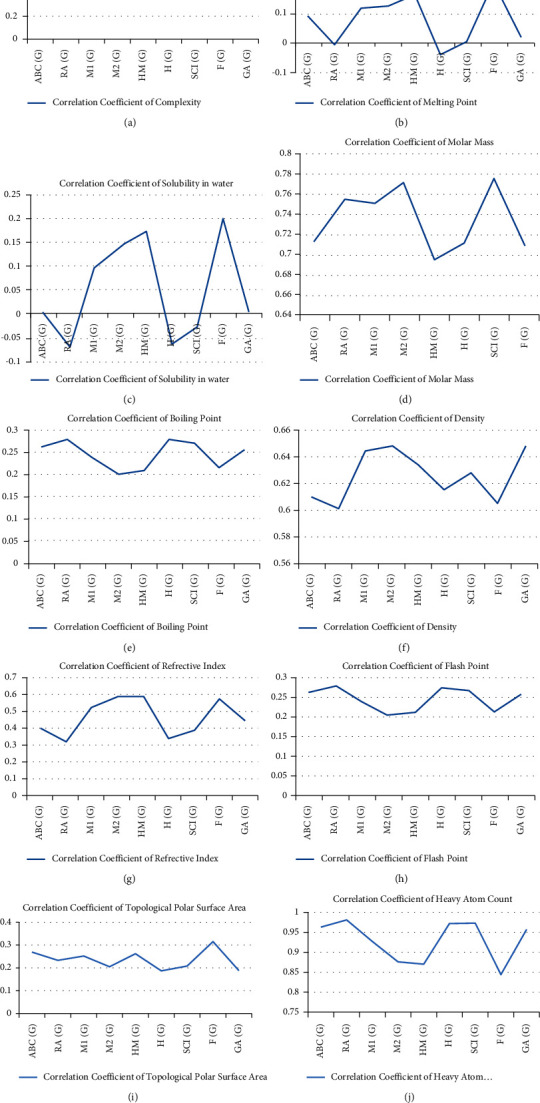
Correlation of physiochemical properties with TIs. (a) Complexity on TI. (b) Melting point on TI. (c) Solubility in water on TI. (d) Molar mass on TI. (e) Boiling point on TI. (f) Density on TI. (g) Refractive index on TI. (h) Flash point on TI. (i) Topological polar surface area on TI. (j) Heavy atom count on TI.

Relation between TIs and physical properties of fungicides is successfully analyzed by imposing QSPR modeling. This sort of analysis can be useful for the model. It is eminent the value of *p* is less than 0.05 and *r* is greater than 0.6. Hence it is concluded that the entire properties given in Tables 3–11 are significant.  Figure 4 depicts the graph.

#### 3.1.1. Regression Models for ABC(*G*)

(10) $$\begin{array}{c} \text{Solubility in water}=86.314+.295\left[\text{ABC}\left(G\right)\right]\text{,}\\ \text{Boiling point}=343.135+8.246\left[\text{ABC}\left(G\right)\right]\text{,}\\ \text{Density}=0.626+.041\left[\text{ABC}\left(G\right)\right]\text{,}\\ \text{Melting point}=49.859+3.180\left[\text{ABC}\left(G\right)\right]\text{,}\\ \text{Molar mass}=125.565+11.392\left[\text{ABC}\left(G\right)\right]\text{,}\\ \text{Flash point}=161.403+4.983\left[\text{ABC}\left(G\right)\right]\text{,}\\ \text{Topological polar surface area}=6.608+2.991\left[\text{ABC}\left(G\right)\right]\text{,}\\ \text{Heavy atom count}=3.597+1.077\left[\text{ABC}\left(G\right)\right]\text{,}\\ \text{Complexity}=-65.744+25.904\left[\text{ABC}\left(G\right)\right]\text{,}\\ \text{Refractive index}=1.386+.014\left[\text{ABC}\left(G\right)\right]\text{.} \end{array}$$

#### 3.1.2. Regression Models for RA(*G*)

(11) $$\begin{array}{c} \text{Solubility in water}=187.306-9.686\left[\text{RA}\left(G\right)\right]\\ \text{Boiling point}=328.548+14.870\left[\text{RA}\left(G\right)\right]\\ \text{Density}=0\;.610+0\;.069\left[\text{RA}\left(G\right)\right]\\ \text{Melting point}=104.598-0.217\left[\text{RA}\left(G\right)\right]\\ \text{Molar mass}=120.460+19.059\left[\text{RA}\left(G\right)\right]\\ \text{Flash point}=152.595+8.984\left[\text{RA}\left(G\right)\right]\\ \text{Topological polar surface area}=11.439+4.395\left[\text{RA}\left(G\right)\right]\\ \text{Heavy atom count}=2.545+1.858\left[\text{RA}\left(G\right)\right]\\ \text{Complexity}=-56.575+41.290\left[\text{RA}\left(G\right)\right]\\ \text{Refractive index}=1.428+.018\left[\text{RA}\left(G\right)\right]\text{.} \end{array}$$

#### 3.1.3. Regression Models for SCI(*G*)

(12) $$\begin{array}{c} \text{Solubility in water}=122.660-3.065\left[\text{SCI}\left(G\right)\right]\\ \text{Boiling Point}=344.671+12.804\left[\text{SCI}\left(G\right)\right]\\ \text{Density}=0.631+.065\left[\text{SCI}\left(G\right)\right]\\ \text{Melting point}=97.816+.435\left[\text{SCI}\left(G\right)\right]\\ \text{Molar mass}=134.489+17.041\left[\text{SCI}\left(G\right)\right]\\ \text{Flash point}=162.321+7.738\left[\text{SCI}\left(G\right)\right]\\ \text{Topological Polar Surface Area}=19.082+3.511\left[\text{SCI}\left(G\right)\right]\\ \text{Heavy Atom Count}=4.041+1.649\left[\text{SCI}\left(G\right)\right]\\ \text{Complexity}=-42.909+38.510\left[\text{SCI}\left(G\right)\right]\\ \text{Refractive index}=1.403+.020\left[\text{SCI}\left(G\right)\right]\text{.} \end{array}$$

#### 3.1.4. Regression Models for GA(*G*)

(13) $$\begin{array}{c} \text{Solubility in water}=83.186+.365\left[\text{GA}\left(G\right)\right]\\ \text{Boiling point}=361.053+5.359\left[\text{GA}\left(G\right)\right]\\ \text{Density}=.667+.029\left[\text{GA}\left(G\right)\right]\\ \text{Melting point}=91.378+.499\left[\text{GA}\left(G\right)\right]\\ \text{Molar mass}=148.492+7.486\left[\text{GA}\left(G\right)\right]\\ \text{Flash point}=172.207+3.239\left[\text{GA}\left(G\right)\right]\\ \text{Topological polar surface area}=25.199+1.396\left[\text{GA}\left(G\right)\right]\\ \text{Heavy atom count}=5.637+.713\left[\text{GA}\left(G\right)\right]\\ \text{Complexity}=-24.023+17.495\left[\text{GA}\left(G\right)\right]\\ \text{Refractive index}=1.390+.010\left[\text{GA}\left(G\right)\right]\text{.} \end{array}$$

#### 3.1.5. Regression Models for *M*_1_(*G*)

(14) $$\begin{array}{c} \text{Solubility in water}=-9.471+0.920\left[M_{1}\left(G\right)\right]\\ \text{Boiling point}=370.704+.975\left[M_{1}\left(G\right)\right]\\ \text{Density}=0\;.677+.006\left[M_{1}\left(G\right)\right]\\ \text{Melting point}=42.770+.535\left[M_{1}\left(G\right)\right]\\ \text{Molar mass}=141.046+1.549\left[M_{1}\left(G\right)\right]\\ \text{Flash point}=178.030+.589\left[M1\left(G\right)\right]\\ \text{Topological polar surface area}=15.411+.364\left[M_{1}\left(G\right)\right]\\ \text{Heavy atom count}=6.361+.135\left[M_{1}\left(G\right)\right]\\ \text{Complexity}=-50.995+3.707\left[M_{1}\left(G\right)\right]\\ \text{Refractive index}=1.355+.002\left[M_{1}\left(G\right)\right]\text{.} \end{array}$$

#### 3.1.6. Regression Models for HM(*G*)

(15) $$\begin{array}{c} \text{Solubility in water}=-60.409+.275\left[\text{HM}\left(G\right)\right]\\ \text{Boiling point}=393.103+.154\left[\text{HM}\left(G\right)\right]\\ \text{Density}=.749+.001\left[\text{HM}\left(G\right)\right]\\ \text{Melting point}=25.196+.138\left[\text{HM}\left(G\right)\right]\\ \text{Molar mass}=154.568+.284\left[\text{HM}\left(G\right)\right]\\ \text{Flash point}=191.549+.093\left[\text{HM}\left(G\right)\right]\\ \text{Topological polar surface area}=17.964+.068\left[\text{HM}\left(G\right)\right]\\ \text{Heavy atom count}=8.695+.023\left[\text{HM}\left(G\right)\right]\\ \text{Complexity}=-30.761+.701\left[\text{HM}\left(G\right)\right]\\ \text{Refractive index}=1.351+.000\left[\text{HM}\left(G\right)\right]\text{.} \end{array}$$

#### 3.1.7. Regression Models for *M*_2_(*G*)

(16) $$\begin{array}{c} \text{Solubility in water}=-20.127+.868\left[M_{2}\left(G\right)\right]\\ \text{Boiling point}=402.839+.585\left[M_{2}\left(G\right)\right]\\ \text{Density}=.786+.004\left[M_{2}\left(G\right)\right]\\ \text{Melting point}=48.750+.410\left[M_{2}\left(G\right)\right]\\ \text{Molar mass}=172.188+1.083\left[M_{2}\left(G\right)\right]\\ \text{Flash point}=197.430+.354\left[M_{2}\left(G\right)\right]\\ \text{Topological polar surface area}=28.830+.208\left[M_{2}\left(G\right)\right]\\ \text{Heavy atom count}=9.699+.089\left[M_{2}\left(G\right)\right]\\ \text{Complexity}=7.449+2.714\left[M_{2}\left(G\right)\right]\\ \text{Refractive index}=1.373+.002\left[M_{2}\left(G\right)\right]\text{.} \end{array}$$

#### 3.1.8. Regression Models for *F*(*G*)

(17) $$\begin{array}{c} \text{Solubility in water}=-98.456+.645\left[F\left(G\right)\right]\\ \text{Boiling point}=386.780+.310\left[F\left(G\right)\right]\\ \text{Density}=0.740+.002\left[F\left(G\right)\right]\\ \text{Melting point}=1.929+.336\left[F\left(G\right)\right]\\ \text{Molar mass}=143.313+.570\left[F\left(G\right)\right]\\ \text{Flash point}=187.734+.187\left[F\left(G\right)\right]\\ \text{Topological polar surface area}=7.378+.163\left[F\left(G\right)\right]\\ \text{Heavy atom count}=8.273+.044\left[F\left(G\right)\right]\\ \text{Complexity}=-52.270+1.387\left[F\left(G\right)\right]\\ \text{Refractive index}=1.340+.001\left[F\left(G\right)\right]\text{.} \end{array}$$

#### 3.1.9. Regression Models for *H*(*G*)

(18) $$\begin{array}{c} \text{Solubility in water}=170.472-8.336\left[H\left(G\right)\right]\\ \text{Boiling point}=339.625+14.367\left[H\left(G\right)\right]\\ \text{Density}=.636+.069\left[H\left(G\right)\right]\\ \text{Melting point}=125.068-2.332\left[H\left(G\right)\right]\\ \text{Molar mass}=136.206+18.255\left[H\left(G\right)\right]\\ \text{Flash point}=159.276+8.682\left[H\left(G\right)\right]\\ \text{Topological polar surface area}=22.495+3.446\left[H\left(G\right)\right]\\ \text{Heavy atom count}=3.821+1.806\left[H\left(G\right)\right]\\ \text{Complexity}=-29.312+40.254\left[H\left(G\right)\right]\\ \text{Refractive index}=1.428+.019\left[H\left(G\right)\right]\text{.} \end{array}$$

### 3.2. Standard Error of Estimate (SEE), Correlation Determination, and Comparison

A measure of variation for an observation calculated around the computed regression line is said to be the standard error estimate. It examines the extent of accuracy of predictions made about the calculated regression line in Table 12.

## 4. Conclusions

It is noted that Randic index RA(*G*) provides high correlated value of heavy atom count at *r* = 0981. *F*(*G*) index provides maximum correlated value for molar mass *r* = 0.776 and complexity *r* = 0.788. No correlation was found between TIs and density, polar surface area, flash point, boiling point, melting point, refractive index, and solubility in water.

In this work, the TIS for fungicides were computed, and they were contrasted with a linear QSPR model. Using the data gathered in this manner, the pharmaceutical industry will be able to create new medications to discover preventative treatments for the aforementioned illness. The variety of topological indicators for these medications is strongly affected by the correlation coefficient. The results offer a technique to evaluate physiochemical features for new discoveries of other disorders and are eye-opening for researchers working on drug science in the pharmaceutical sector.

## Figures and Tables

**Table 1:** The TIs values of candidate drugs.

Names of drug	ABC(*G*)	RA(*G*)	*M*_1_(*G*)	*M*_2_(*G*)	HM(*G*)	*H*(*G*)	SCI(*G*)	*F*(*G*)	GA(*G*)
Paclobutrazol	15.40229	9.376029	102	116	508	8.852381	9.613811	276	19.96016
Tebuconazole	16.20668	9.800443	108	123	548	9.216667	10.03661	302	20.79001
Flutriafol	17.1846	10.59317	116	137	580	10.2381	11.04456	306	23.27251
Myclobutanil	14.34883	9.137977	94	107	458	8.819048	9.34952	244	19.34277
Propiconazole	17.08729	10.62696	116	137	580	10.28571	11.06044	306	23.29099
Prothioconazole	16.62446	9.935071	116	140	608	9.45	10.38075	328	21.98206
Epoxiconazole	17.63074	10.71518	124	154	640	10.45714	11.38356	332	24.46225
Triadimefon	15.40229	9.376029	102	116	508	8.852381	9.613811	276	19.96016
Cyproconazole	15.77039	9.593172	108	129	548	9.238095	10.04456	290	21.27251
Flusilazole	17.22504	10.57634	116	136	578	10.20476	11.03074	306	23.2321
Hexaconazole	15.12489	9.548661	100	115	494	9.152381	9.757768	264	20.20879
Flucanozole	17.24621	10.5663	116	135	576	10.18571	11.02206	306	23.20537
Voriconazole	19.48426	11.98945	131	154	657	11.47143	12.40002	349	25.98436

**Table 2:** Physical properties of drugs.

Names of drugs	Solubility in water (mg/L at 20°C)	Boiling point (°C)	Density (g/cm^3^)	Melting point (°C)	Molar mass (g/mol)	Flash point (°C)	Topological polar surface area (Å^2^)	Heavy atom count	Complexity	Refractive index
Paclobutrazol	22.9	460.9 ± 55	1.23	165	293.8	232.6 ± 31.5	50.9	20	300	1.58
Tebuconazole	36	476.9 ± 55	1.249	102.4	307.82	242.2 ± 31.5	50.9	21	326	1.58
Flutriafol	130	506.5 ± 60	1.3	130	301.29	260.1 ± 32.9	50.9	22	365	1.6
Myclobutanil	142	465.2 ± 55	1.2	65	288.78	235.2 ± 31.5	54.5	20	345	1.589
Propiconazole	100	480.0 ± 55	1.39	−23	342.22	244.1 ± 31.5	49.2	22	377	1.623
Prothioconazole	300	486.7 ± 55	1.36	141.5	344.2	248.2 ± 31.5	80	21	458	1.698
Epoxiconazole	7.1	463.1 ± 55	1.374	134	329.76	233.9 ± 31.5	43.2	23	421	1.659
Triadimefon	64	441.9 ± 55	1.22	82	293.75	221.0 ± 31.5	57	20	338	1.579
Cyproconazole	140	479.1 ± 55	1.32	106.2	291.77	243.6 ± 31.5	50.9	20	331	1.633
Flusilazole	41.9	392.5 ± 52	1.2	52	315.39	191.2 ± 30.7	30.7	22	333	1.563
Hexaconazole	18	490.3 ± 55	1.3	111	314.21	250.3 ± 31.5	50.9	20	308	1.549
Flucanozole		579.8 ± 60	1.5	138–140	306.271	304.4 ± 32.9	82	22	358	1.683
Voriconazole		508.6 ± 60	1.4 ± 0.1	127–130	349.3	261.4 ± 32.9	77	25	448	1.617

**Table 3:** Statistical parameters used in QSPR model for ABC(*G*).

Physiochemical property	*N*	*A*	*b*	*r*	*r* ^2^	*F*	*P*
Solubility in water	11	86.314	0.295	0.004	0.000	0.000	0.992
Boiling point	13	343.135	8.246	0.261	0.068	0.804	0.389
Density	13	0.626	0.041	0.610	0.372	6.514	0.027
Melting point	13	49.859	3.180	0.087	0.007	0.083	0.779
Molar mass	13	125.565	11.392	0.720	0.519	11.863	0.005
Flash point	13	161.403	4.983	0.261	0.068	0.803	0.389
Topological polar surface area	13	6.608	2.991	0.269	0.072	0.857	0.374
Heavy atom count	13	3.597	1.077	0.963	0.927	139.079	0.000
Complexity	13	−65.744	25.904	0.684	0.467	9.654	0.010
Refractive index	13	1.386	0.014	0.399	0.159	2.086	0.177

**Table 4:** Statistical parameters used in QSPR model for RA(*G*).

Physiochemical property	*N*	*A*	*b*	*r*	*r* ^2^	*F*	*P*
Solubility in water	11	187.306	−9.686	0.067	0.004	0.040	0.846
Boiling point	13	328.548	14.870	0.278	0.077	0.920	0.358
Density	13	0.610	0.069	0.601	0.361	6.218	0.030
Melting point	13	104.598	−0.217	0.003	0.000	0.000	0.991
Molar mass	13	120.460	19.059	0.712	0.506	11.280	0.006
Flash point	13	152.595	8.984	0.278	0.077	0.919	0.358
Topological polar surface area	13	11.439	4.395	0.233	0.054	0.633	0.443
Heavy atom count	13	2.545	1.858	0.981	0.961	274.362	0.000
Complexity	13	−56.575	41.290	0.643	0.414	7.770	0.018
Refractive index	13	1.428	0.018	0.312	0.098	1.190	0.299

**Table 5:** Statistical parameters used in QSPR model for SCI(*G*).

Physiochemical property	*N*	*A*	*b*	*r*	*r* ^2^	*F*	*P*
Solubility in water	11	122.660	−3.065	0.026	0.001	0.006	0.940
Boiling point	13	344.671	12.804	0.267	0.072	0.848	0.377
Density	13	0.631	0.065	0.628	0.395	7.173	0.021
Melting point	13	97.816	0.435	0.008	0.000	0.001	0.980
Molar mass	13	134.489	17.041	0.711	0.506	11.276	0.006
Flash point	13	162.321	7.738	0.267	0.072	0.847	0.377
Topological polar surface area	13	19.082	3.511	0.208	0.043	0.499	0.494
Heavy atom count	13	4.041	1.649	0.973	0.947	197.308	0.000
Complexity	13	−42.909	38.510	0.671	0.450	9.011	0.012
Refractive index	13	1.403	0.020	0.383	0.147	1.893	0.196

**Table 6:** Statistical parameters used in QSPR model for GA(*G*).

Physiochemical property	*N*	*A*	*b*	*r*	*r* ^2^	*F*	*P*
Solubility in water	11	83.186	0.365	0.007	0.000	0.000	0.983
Boiling point	13	361.053	5.359	0.254	0.065	0.761	0.402
Density	13	0.667	0.029	0.645	0.415	7.818	0.017
Melting point	13	91.378	0.499	0.020	0.000	0.005	0.947
Molar mass	13	148.492	7.486	0.710	0.504	11.194	0.007
Flash point	13	172.207	3.239	0.254	0.065	0.761	0.402
Topological polar surface area	13	25.199	1.396	0.188	0.035	0.404	0.538
Heavy atom count	13	5.637	0.713	0.957	0.915	119.159	0.000
Complexity	13	−24.023	17.495	0.693	0.480	10.149	0.009
Refractive index	13	1.390	0.010	0.439	0.193	2.627	0.133

**Table 7:** Statistical parameters used in QSPR model for *M*_1_(*G*).

Physiochemical property	*N*	*A*	*b*	*r*	*r* ^2^	*F*	*P*
Solubility in water	11	−9.471	0.920	0.098	0.010	0.087	0.775
Boiling point	13	370.704	0.975	0.237	0.056	0.656	0.435
Density	13	0.677	0.006	0.644	0.415	7.806	0.017
Melting point	13	42.770	0.535	0.112	0.013	0.140	0.716
Molar mass	13	141.046	1.549	0.754	0.568	14.480	0.003
Flash point	13	178.030	0.589	0.237	0.056	0.657	0.435
Topological polar surface area	13	15.411	0.364	0.252	0.064	0.746	0.406
Heavy atom count	13	6.361	0.135	0.927	0.860	67.373	0.000
Complexity	13	−50.995	3.707	0.753	0.567	14.383	0.003
Refractive index	13	1.355	0.002	0.517	0.267	4.010	0.070

**Table 8:** Statistical parameters used in QSPR model for HM(*G*).

Physiochemical property	*N*	*A*	*b*	*r*	*r* ^2^	*F*	*P*
Solubility in water	11	−60.409	0.275	0.174	0.030	0.280	0.610
Boiling point	13	393.103	0.154	0.209	0.044	0.504	0.493
Density	13	0.749	0.001	0.634	0.402	7.385	0.020
Melting point	13	25.196	0.138	0.161	0.026	0.293	0.599
Molar mass	13	154.568	0.284	0.772	0.596	16.218	0.002
Flash point	13	191.549	0.093	0.209	0.044	0.505	0.492
Topological polar surface area	13	17.964	0.068	0.262	0.069	0.813	0.387
Heavy atom count	13	8.695	0.023	0.870	0.757	34.253	0.000
Complexity	13	−30.761	0.701	0.795	0.633	18.934	0.001
Refractive index	13	1.351	0.000	0.585	0.342	5.709	0.036

**Table 9:** Statistical parameters used in QSPR model for *M*_2_(*G*).

Physiochemical property	*N*	*A*	*b*	*r*	*r* ^2^	*F*	*P*
Solubility in water	11	−20.127	0.868	0.142	0.020	0.185	0.677
Boiling point	13	402.839	0.585	0.203	0.041	0.473	0.506
Density	13	0.786	0.004	0.648	0.420	7.959	0.017
Melting point	13	48.750	0.410	0.122	0.015	0.167	0.690
Molar mass	13	172.188	1.083	0.751	0.564	14.214	0.003
Flash point	13	197.430	0.354	0.203	0.041	0.474	0.505
Topological polar surface area	13	28.830	0.208	0.205	0.042	0.482	0.502
Heavy atom count	13	9.699	0.089	0.876	0.768	36.388	0.000
Complexity	13	7.449	2.714	0.785	0.617	17.696	0.001
Refractive index	13	1.373	0.002	0.584	0.342	5.706	0.036

**Table 10:** Statistical parameters used in QSPR model for *F*(*G*).

Physiochemical property	*N*	*A*	*b*	*r*	*r* ^2^	*F*	*P*
Solubility in water	11	−98.456	0.645	0.202	0.041	0.381	0.552
Boiling point	13	386.780	0.310	0.211	0.044	0.512	0.489
Density	13	0.740	0.002	0.605	0.366	6.357	0.028
Melting point	13	1.929	0.336	0.197	0.039	0.443	0.519
Molar mass	13	143.313	0.570	0.776	0.602	16.662	0.002
Flash point	13	187.734	0.187	0.211	0.045	0.513	0.489
Topological polar surface area	13	7.378	0.163	0.315	0.099	1.211	0.295
Heavy atom count	13	8.273	0.044	0.844	0.713	27.269	0.000
Complexity	13	−52.270	1.387	0.788	0.620	17.985	0.001
Refractive index	13	1.340	0.001	0.572	0.327	5.336	0.041

**Table 11:** Statistical parameters used in QSPR model for *H*(*G*).

Physiochemical property	*N*	*A*	*b*	*r*	*r* ^2^	*F*	*P*
Solubility in water	11	170.472	−8.336	0.063	0.004	0.035	0.855
Boiling point	13	339.625	14.367	0.274	0.075	0.891	0.365
Density	13	0.636	0.069	0.615	0.378	6.693	0.025
Melting point	13	125.068	−2.332	0.038	0.001	0.016	0.901
Molar mass	13	136.206	18.255	0.695	0.483	10.290	0.008
Flash point	13	159.276	8.682	0.274	0.075	0.891	0.365
Topological polar surface area	13	22.495	3.446	0.187	0.035	0.397	0.542
Heavy atom count	13	3.821	1.806	0.972	0.946	190.910	0.000
Complexity	13	−29.312	40.254	0.640	0.409	7.625	0.019
Refractive index	13	1.428	0.019	0.332	0.110	1.365	0.267

**Table 12:** Standard error of estimate.

Topological indices	*Standard error of estimate*									
	Solubility in water	Boiling point	Density	Melting point	Molar Mass	Flash point	Topological polar surface area	Heavy atom count	Complexity	Refractive index
ABC(*G*)	90.3417	42.7913	0.075526	51.3723	15.38515	25.8647	15.0241	0.425	38.783	0.044068
RA(*G*)	90.1415	42.5820	0.076173	51.5652	15.58503	25.7384	15.1681	0.308	40.682	0.045658
*M*_1_(*G*)	89.9110	43.0604	0.072886	51.2414	14.57363	26.0265	15.0952	0.588	34.984	0.041146
*M*_2_(*G*)	89.4259	43.4031	0.072591	51.1779	14.65046	26.2331	15.2673	0.756	32.902	0.039002
HM(*G*)	88.9703	43.3450	0.073716	50.8925	14.10078	26.1981	15.0522	0.774	32.215	0.038999
*H*(*G*)	90.1651	42.6330	0.075143	51.5279	15.94348	25.7689	15.3246	0.366	40.841	0.045334
SCI(*G*)	90.3127	42.7115	0.074144	51.5640	15.58663	25.8162	15.2560	0.361	39.401	0.044396
*F*(*G*)	88.4891	43.3293	0.075867	50.5571	13.98699	26.1888	14.8049	0.841	32.738	0.039441
GA(*G*)	90.3398	42.8683	0.072862	51.5548	15.61538	25.9106	15.3196	0.456	38.327	0.043184

## Data Availability

All the data used to support the findings of the study are included within the article.
